# Measuring eccentric hamstring strength in senior athletes: reference values, age and sex associations, and limb asymmetry

**DOI:** 10.3389/fspor.2026.1933413

**Published:** 2026-10-14

**Authors:** Lee J. Hinkle, James D. Mead, Emma A. Merrill, Joshua K. Sponbeck, Ron Hager, A. Wayne Johnson

**Affiliations:** 1 Neuromuscular Physiology Lab, Department of Kinesiology, College of Health and Human Sciences, Kansas State University, Manhattan, KS, United States; 2 Rehabilitation Imaging & Performance Laboratory, Department of Exercise Sciences, College of Life Sciences, Brigham Young University, Provo, UT, United States; 3 Department of Orthopaedic Surgery Operations, University of Utah School of Medicine, University of Utah, Salt Lake City, UT, United States; 4 Department of Athletic Training and Occupational Therapy, Dumke College of Health Professions, Weber State University, Ogden, UT, United States; 5 Department of Gerontology, College of Family, Home and Social Sciences, Brigham Young University, Provo, UT, United States

**Keywords:** aging athletes, eccentric hamstring strength, injury risk, muscle asymmetry, Nordic hamstring curl

## Abstract

**Background:**

Participation in Masters sport has increased; however, reference eccentric hamstring strength values are unavailable. Instrumented Nordic hamstring exercise (NHE) testing is used in younger athletes, but reports for senior athletes are lacking.

**Purpose:**

Establish sex-stratified reference values for senior athletes. Secondary aims examined associations with age, sex, and inter-limb asymmetry.

**Methods:**

This study included 203 senior athletes (100 female) and measured eccentric hamstring strength during testing. Absolute peak force, relative peak force, and limb asymmetry were calculated.

**Results:**

Mean unilateral absolute peak force was 156 N in females and 242 N in males, corresponding to relative values of 2.46 *N*/kg and 2.99 *N*/kg, respectively. Males demonstrated greater absolute peak force (mean difference: 85.6 95% CI 70.2 to 100.9; *d* = 1.54, 95% CI 1.22 to 1.85) and greater relative peak force (mean difference: 0.53*N*/kg, 95% CI 0.34 to 0.73; *d* = 0.76, 95% CI 0.47 to 1.04). Increasing age was associated with lower absolute peak force (−2.10 *N*/year, 95% CI −3.09 to −1.11) and lower relative peak force (−0.020 *N*/kg/year, 95% CI −0.033 to −0.008). Mean asymmetry was 11.6 ± 8.8% (75th percentile: 16.1%). Asymmetry was not significantly associated with age or sex.

**Conclusions:**

This study provides the first reference eccentric hamstring strength values for senior athletes. Male athletes demonstrated greater absolute and relative strength than females, whereas increasing age was associated with lower strength despite high physical activity levels. Common asymmetry thresholds may overclassify high-functioning senior athletes as having concerning inter-limb asymmetry, highlighting the need for population-specific interpretation.

## 1 Introduction

Participation in organized sport among senior Masters athletes (aged ≥ 50 years) has increased substantially in recent decades (1–3). These athletes represent a rapidly growing population that maintains high levels of training and competition into later life. Hamstring injuries are common in sports involving running and sprinting (4, 5), and advancing age may further increase injury risk (6). Programs incorporating the Nordic hamstring exercise (NHE) have been shown to reduce hamstring injury rates by up to 51% (7), supporting the relevance of eccentric hamstring strength in injury-prevention and performance settings. Accordingly, eccentric hamstring strength is frequently assessed using instrumented NHE testing in athletes participating in sprint- and running-based sports, including football codes and track and field (8–11), as well as skiers (12) using instrumented Nordic hamstring exercise testing due to its relevance to performance and hamstring injury risk in athletic cohorts (13, 14). Although reference values for eccentric hamstring strength have been reported in elite junior (12, 15), collegiate (16), and professional athletes (8, 10), no reference values are available for older athletic populations. This absence is especially relevant because age-related declines in muscle strength are well documented, yet strength profiles in highly active older adults may differ from less physically active older adults due to sustained training exposure (17).

Further, hamstring strength is commonly reported in absolute terms (*N*), despite substantial inter-individual variation in body mass (18). Reporting relative strength (*N*/kg) may improve interpretability across individuals and between sexes, yet comparative data incorporating both absolute and relative metrics in senior athletes remain limited. Similarly, limb asymmetry thresholds are frequently cited in applied settings (19, 20), although age- and sex-specific reference distributions in older athletes are not well characterized.

Accordingly, the primary purpose of this study was to establish sex-stratified reference values for eccentric hamstring strength in competitive senior athletes aged 50 years and older. Absolute and relative peak force percentiles were calculated to facilitate clinical and performance benchmarking within this population. Secondary aims were to examine cross-sectional associations between age and peak strength, to quantify sex differences in absolute and body mass–normalized force, and to characterize the distribution of hamstring asymmetry. Collectively, these analyses were intended to support interpretation of eccentric hamstring strength measures obtained during instrumented Nordic hamstring exercise testing in older athletic populations.

## 2 Methods

### 2.1 Study design and setting

This cross-sectional observational study was conducted at the Huntsman World Senior Games in 2022 and 2023, where more than 11,000 athletes meet annually to compete in more than 40 sports and events. Data were collected on-site within a designated performance assessment area. The study was approved by the Institutional Review Board (IRB #2022-368), and all participants provided written informed consent prior to participation.

### 2.2 Participants

A total of 203 participants (100 female) provided complete demographic and hamstring strength data across both years. Eligible individuals were registered Huntsman World Senior Games athletes aged ≥50 years who were able to safely perform the Nordic hamstring exercise. The analytic sample across a broad range of Huntsman World Senior Games sports, with representation from team, racquet, endurance, and individual sports. Analyses were restricted to participants with complete demographic and hamstring strength data. Participant characteristics are presented in Table 1. Because the sample consisted of highly active senior athletes, these findings should be interpreted as reference values for older athletic populations rather than the general population.

**Table 1:** Descriptive demographic measures of the cohort, all variables reported in mean ± sd, except moderate-vigorous physical activity (MVPA) weekly mins, which is reported in median [Q1-Q3 range].

Sex	Age Range	*N*	Age	Height (cm)	Weight (kg)	MVPA Weekly Mins	Unilateral Peak Force NHE (*N*)
Female	50–59	22	56.9 ± 1.7	165.6 ± 7.1	65.8 ± 10.2	360.0 [288.8, 690.0]	169.1 ± 31.7
Female	60–69	51	65.5 ± 2.7	166.0 ± 7.2	64.0 ± 8.9	570.0 [367.5, 1,080.0]	158.7 ± 44.6
Female	70+	27	75.1 ± 4.7	164.9 ± 9.4	64.3 ± 11.6	540.0 [390.0, 1,020.0]	141.0 ± 40.4
Male	50–59	5	55.2 ± 2.3	180.3 ± 10.8	84.0 ± 16.0	660.0 [480.0, 720.0]	252.6 ± 46.1
Male	60–69	46	65.0 ± 2.6	178.2 ± 9.1	82.2 ± 10.5	960.0 [600.0, 1,770.0]	256.1 ± 74.9
Male	70+	52	76.6 ± 5.3	178.0 ± 8.3	80.0 ± 11.6	735.0 [510.0, 1,245.0]	228.1 ± 57.5

### 2.3 Questionnaire measures

Participants completed a brief questionnaire capturing demographic information and physical activity using the International Physical Activity Questionnaire–Short Form (IPAQ-SF).

### 2.4 Hamstring strength testing protocol

Eccentric hamstring strength was assessed using the NordBord system (VALD Performance, Newstead, QLD, Australia) following a standardized protocol (9). Participants completed a self-selected warm-up (5–10 min of light activity or stretching), followed by three submaximal Nordic hamstring repetitions at approximately 50%, 75%, and 90% perceived effort. This was followed by three maximal-effort trials, each separated by 60 s of rest.

During each repetition, participants were instructed to resist forward descent as long as possible. Testing was discontinued if participants reported pain or cramping. Peak eccentric hamstring force (*N*) for each limb was recorded during each maximal trial. Reliability testing in a separate sample using the same testing protocol and equipment demonstrated high within-session reliability for peak eccentric force across three maximal trials (*n* = 27; ICC[A,1] = 0.88, 95% CI: 0.78–0.94).

### 2.5 Outcome variables

Peak eccentric hamstring strength was defined as the highest force value (*N*) recorded from either limb across all maximal Nordic hamstring exercise trials. Relative peak force (*N*/kg) was calculated by normalizing peak force to body mass. Hamstring asymmetry was quantified using the NordBord-derived limb imbalance percentage, with absolute asymmetry defined as the absolute value of the imbalance percentage to represent the magnitude of between-limb difference independent of direction.

### 2.6 Statistical analysis

All analyses were conducted in R (21). Descriptive statistics were used to generate reference values for absolute (*N*) and relative (*N*/kg) peak force, as well as asymmetry. Sex-stratified percentiles (10th, 25th, 50th, 75th, and 90th) were calculated for the overall sample and within age bands (50–59, 60–69, and ≥70 years).

Sex differences in absolute peak force, relative peak force, and asymmetry were evaluated using Welch's independent two-sample *t*-tests, with effect sizes reported as Cohen's d and corresponding 95% confidence intervals. Cohen's d values of 0.2, 0.5, and 0.8 were interpreted as small, moderate, and large effects, respectively (35).

Linear regression models were used to examine associations between age, sex, and outcomes including absolute peak force, relative peak force, and asymmetry. Age was modeled as a continuous predictor and sex as a categorical predictor. Model assumptions were assessed using residual, Q-Q, scale-location, and leverage diagnostic plots to evaluate approximate normality, linearity, homoscedasticity, and potentially influential observations. Following conventional diagnostic criteria, observations with absolute studentized residuals >3 were considered potentially unusual and examined further. Cook's distance was also examined, and a sensitivity analysis excluding observations with large studentized residuals did not materially alter the regression findings (36, 37). Statistical significance was set at *α* = 0.05; however, given the descriptive aims of the study, interpretation emphasized effect sizes and confidence intervals over *p*-values.

## 3 Results

### 3.1 Participant characteristics

A total of 203 senior athletes (100 female, 103 male) completed testing across the 2022 and 2023 Huntsman World Senior Games. Mean participant age was 68.3 years (range: 53–91 years). Participant characteristics are presented in Table 1. Participants reported high levels of physical activity, with a median of 660 min/week of moderate-to-vigorous physical activity (IQR: 398–1200 min/week). One implausible physical activity value was excluded from these descriptive summaries.

### 3.2 Peak hamstring strength and sex differences

Across the full sample, median unilateral absolute peak hamstring force was 186.25 N (range: 57.25–496.75 N), and median unilateral relative peak force was 2.66 *N*/kg (range: 0.97–5.22 *N*/kg). Sex-stratified values are presented in Table 2. Median absolute peak force was 150 N in females and 240 N in males, while median relative peak force was 2.42 *N*/kg and 3.04 *N*/kg, respectively. Inferential comparisons were based on group means. Males demonstrated greater absolute peak force, with a very large effect size (Cohen's *d* = 1.54, 95% CI 1.22 to 1.85; mean difference = 85.6 N, 95% CI 70.2 to 100.9; *p* < 0.001). Males also demonstrated greater relative peak force, with a moderate effect size (Cohen's *d* = 0.76, 95% CI 0.47 to 1.04; mean difference = 0.53 *N*/kg, 95% CI 0.34 to 0.73; *p* < 0.001).

**Table 2:** Sex- and age-stratified reference values for unilateral eccentric hamstring strength. Panel A presents absolute peak force (*N*), and Panel B presents peak force normalized to body mass (*N*/kg). Values are reported as mean ± SD and the 10th (P10), 25th (P25), 50th (P50), 75th (P75), and 90th (P90) percentiles.

A. Absolute peak force (*N*)								
Sex	Age group	*n*	Mean ± SD	P10	P25	P50	P75	P90
Female	50–59	22	169.1 ± 31.7	125.2	150.2	167.9	190.9	207.3
Female	60–69	51	158.7 ± 44.6	114.2	132.1	147	178.4	205.2
Female	≥70	27	141.0 ± 40.4	100.7	114	137.8	158.8	187.1
Male	50–59	5^a^	252.6 ± 46.1	215.4	231.5	233.5	269	302.1
Male	60–69	46	256.1 ± 74.9	173.9	213.4	240.6	282.4	350.4
Male	≥70	52	228.1 ± 57.5	157.5	183.8	240.1	253.8	299.2

B. Relative peak force (*N*/kg)								
Sex	Age group	*n*	Mean ± SD	P10	P25	P50	P75	P90
Female	50–59	22	2.60 ± 0.50	1.88	2.31	2.53	2.95	3.27
Female	60–69	51	2.53 ± 0.75	1.65	2.01	2.41	2.84	3.69
Female	≥70	27	2.22 ± 0.57	1.48	1.88	2.37	2.53	2.88
Male	50–59	5^a^	3.05 ± 0.57	2.59	2.77	2.87	3.18	3.65
Male	60–69	46	3.14 ± 0.82	2.11	2.66	3.2	3.53	4.1
Male	≥70	52	2.86 ± 0.65	2.12	2.38	2.92	3.32	3.63

a Age-stratified reference values from smaller subgroups should be interpreted cautiously, particularly percentile estimates at the distributional extremes.

### 3.3 Association between Age and peak strength

#### 3.3.1 Absolute peak force

Age was associated with lower absolute peak force (−2.10 N per year, 95% CI −3.09 to −1.11, *p* < 0.001). Male sex was associated with substantially greater absolute strength (+94.41 N, 95% CI 79.11 to 109.70, *p* < 0.001). The model explained a moderate proportion of variance (*R^2^* = 0.424; adjusted *R^2^* = 0.418). Expressed practically, a 10-year increase in age corresponded to an approximate 21 N reduction in absolute peak force.

#### 3.3.2 Relative peak force

Age was associated with lower relative strength (−0.020 *N*/kg per year, 95% CI −0.033 to −0.0075, *p* = 0.002). Male sex was associated with greater relative strength (+0.62 *N*/kg, 95% CI 0.42 to 0.82, *p* < 0.001). The model explained less variance than the absolute model (*R^2^* = 0.167; adjusted *R^2^* = 0.159). A 10-year increase in age corresponded to an approximate 0.20 *N*/kg reduction in relative peak force.

### 3.4 Hamstring asymmetry

Mean absolute asymmetry across the full sample was 11.6 ± 8.8%, with the 75th percentile at 16.1% and a maximum observed value of 42.0%. Sex-stratified asymmetry percentiles are presented in Table 3. Mean asymmetry was 12.1 ± 9.0% in females and 11.1 ± 8.7% in males. Absolute asymmetry did not differ significantly between sexes (*t* = 0.79, *p* = 0.43). Asymmetry was not significantly associated with age (−0.053 percentage points per year, 95% CI −0.216 to 0.111, *p* = 0.53), and sex was not a significant predictor (*p* = 0.56). The model explained negligible variance (*R^2^* = 0.005). Sex comparisons and regression estimates across all outcomes are summarized in Table 4.

**Table 3:** Sex-stratified hamstrings limb asymmetry percentage.

Sex	*N*	Mean	SD	10th	25th	Median	75th	90th
Female	100	12.1	9	1.7	5.1	10.4	17.1	26.4
Male	103	11.1	8.7	1	4.3	9.1	15.7	22.4

**Table 4:** Summary of inferential analyses.

A. Sex differences				
Outcome	Mean difference (Male−Female)	95% CI	Cohen's d (95% CI)	*p*
Absolute peak force (*N*)	85.6	70.2 to 100.9	1.54 (1.22 to 1.85)	<0.001
Relative peak force (*N*/kg)	0.53	0.34 to 0.73	0.76 (0.47 to 1.04)	<0.001
Absolute asymmetry (%)	−0.98	−3.43 to 1.47	0.11 (−0.16 to 0.39)	*0* *.* *43*

B. Associations from linear regression models				
	Predictor	Estimate	95% CI	*p*
Absolute peak force (*N*)	Age, per year	−2.10	−3.09 to −1.11	<0.001
	Male sex	+94.41	79.11 to 109.70	<0.001
Relative peak force (*N*/kg)	Age, per year	−0.020	−0.033 to −0.0075	0.002
	Male sex	+0.62	0.42 to 0.82	<0.001
Absolute asymmetry (%)	Age, per year	−0.052	−0.216 to 0.112	0.53
	Male sex	−0.762	−3.31 to 1.79	0.556

Mean differences in Panel A are expressed as male minus female. Regression coefficients for sex in Panel B represent males relative to females. CI, confidence interval.

Summary of inferential analyses for eccentric hamstring strength and inter-limb asymmetry. Panel A presents sex comparisons using Welch's independent two-sample *t*-tests, with mean differences, 95% confidence intervals, Cohen's d, and *p*-values. Panel B presents linear regression estimates for associations of age and sex with absolute peak force, relative peak force, and asymmetry.

## 4 Discussion

This study established sex-stratified reference values and percentile distributions for eccentric hamstring strength in competitive senior athletes aged ≥50 years using instrumented Nordic hamstring testing. Males demonstrated substantially greater absolute peak force than females, with smaller but persistent differences after normalization to body mass. Increasing age was associated with lower absolute and relative peak force, whereas asymmetry was not associated with age or sex. Collectively, these findings provide reference data to support interpretation of eccentric hamstring strength assessments in older athletic populations.

### 4.1 Reference values and sex differences

Most published Nordic hamstring exercise (NHE) strength datasets are derived from adolescent or young adult athletic cohorts, frequently at elite or professional levels (18, 22, 23), with comparatively little representation of older adults. The present study is the first to report eccentric hamstring strength values in a cohort of senior athletes aged over 50 years. Although participants in the present cohort reported high levels of physical activity, the sample included athletes from a broad range of sports and competitive standards.

Median unilateral peak force was 240 N in males and 150 N in females, corresponding to relative values of 3.04 *N*/kg and 2.42 *N*/kg, respectively. These values were substantially lower than those reported in younger elite and professional athletic cohorts. For example, one group reported eccentric knee flexor strength values of approximately 343–388 N and relative values of 3.65–4.03 *N*/kg across elite, subelite, and under-19 rugby players (22). Similarly, Opar et al. (13) reported mean eccentric hamstring strength values of approximately 301–330 N and relative values of 3.81–4.18 *N*/kg in elite Australian football players. In British track and field athletes, Giakoumis et al. (8) reported average peak force values of 418 N in males and 319 N in females, with relative values approaching 5.0 *N*/kg in both sexes. Professional soccer cohorts assessed using the NHE have also demonstrated average forces exceeding 400 N in some settings (10). Even adolescent cohorts commonly demonstrate relative force values near or above 4.0 *N*/kg by late adolescence (23).

Sex differences persisted after normalization to body mass, suggesting that body mass alone does not fully explain the observed disparity in eccentric hamstring strength. Although some studies have reported attenuation of sex differences after normalization procedures (8), persistent sex effects have also been observed in athletic NHE cohorts (12). Potential contributors may include differences in height, lean mass distribution, limb dimensions, muscle architecture, tendon properties, and neuromuscular characteristics, although these mechanisms were not directly assessed in the present study. In older females specifically, menopause-related reductions in muscle mass and neuromuscular function may also contribute (24, 25).

Future work should extend NHE assessment across the adult lifespan and into less physically active populations to better characterize how ageing, training status, and sport participation influence eccentric hamstring strength across later life.

### 4.2 Age associations

Despite the high activity levels observed in this cohort, increasing age remained independently associated with lower eccentric hamstring strength. This finding suggests that regular athletic participation and high volumes of physical activity do not fully prevent age-related reductions in muscle function, even among senior athletes. However, the magnitude of decline observed in the present study was relatively modest, which may indicate partial attenuation of age-related strength loss through continued training and sport participation. This interpretation is consistent with broader ageing literature demonstrating that physically active older adults generally retain greater muscle strength and functional capacity than sedentary peers, despite ongoing physiological decline (26, 27). Eccentric strength also appears relatively better preserved with ageing than concentric strength, particularly among physically active individuals and those performing regular resistance or eccentric training (17, 28), which may partially explain the maintenance of eccentric hamstring function despite advanced age in the present cohort.

The present findings also align with performance data from masters athletes, where advancing age is associated with progressive reductions in sprint performance, muscle strength, and power output, even among highly trained individuals (3). However, some evidence suggests these declines may be less steep than previously estimated, particularly among highly active older adults (29–31). Interpretation of the present findings should nevertheless remain cautious because the analysis was cross-sectional rather than longitudinal, and the observed association between age and strength may not reflect within-person decline over time. Future longitudinal work is needed to determine whether continued participation in masters sport or eccentric-focused training alters trajectories of hamstring strength decline across later life.

### 4.3 Asymmetry findings

Inter-limb asymmetry during Nordic hamstring testing has received considerable attention within hamstring injury-risk literature. In younger athletic cohorts, greater eccentric strength asymmetry has been associated with increased hamstring strain injury risk. For example, Bourne et al. (22) reported that between-limb imbalances of 15% and 20% were associated with 2.4-fold and 3.4-fold greater hamstring injury risk, respectively, in elite rugby players. These findings have contributed to the widespread use of asymmetry thresholds of approximately 10%–15% within screening and return-to-sport settings.

However, the evidentiary basis supporting universal asymmetry thresholds across populations remains uncertain. Parkinson et al. (19) specifically cautioned against the use of arbitrary asymmetry cut-points, noting that commonly used thresholds are not robustly supported across the literature. The present findings are broadly consistent with the literature examining asymmetry in older adults. In the current cohort, mean hamstring asymmetry was 11.6 ± 8.8%, with 25% of participants exceeding 16% asymmetry despite continued participation in competitive sport and high levels of habitual physical activity. Similar asymmetry magnitudes have been reported in healthy older adults. Carabello et al. (32) reported knee extensor asymmetry values ranging from approximately 10%–15% across middle-aged and older cohorts, while Daneshjoo et al. (33) observed mean hamstring asymmetry values of approximately 9% in older non-fallers. Although direct comparisons between muscle groups and testing methodologies should be made cautiously, these studies collectively suggest that asymmetry values exceeding commonly cited 10% thresholds may frequently occur in otherwise high-functioning older adults.

Collectively, these findings support recommendations that asymmetry values should be interpreted within population-specific contexts rather than relying on universal cut-points derived primarily from younger athletic cohorts (19, 34).

### 4.4 Methodological considerations

Several strengths of the present study should be acknowledged. This study provides the first sex-stratified reference data for eccentric hamstring strength in competitive senior athletes aged ≥50 years. The sample included athletes across a broad age range (53–91 years) and incorporated both absolute and body mass–normalized force metrics, allowing for comparison across sexes and descriptive evaluation across age groups. Additionally, testing was conducted using a standardized instrumented Nordic hamstring protocol with demonstrated within-session reliability.

This study also has limitations. The cross-sectional design precludes interpretation of longitudinal age-related decline, and observed associations between age and peak force should not be interpreted as within-person changes over time. Participants were highly active competitive senior athletes recruited from the Huntsman World Senior Games; therefore, these findings may not generalize to less active older adults or non-athletic populations. Because analyses were restricted to participants with complete demographic and hamstring strength data, selection bias cannot be excluded; sport participation data were unavailable for excluded participants, precluding assessment of differential exclusion by sport. Some age- and sex-stratified subgroups were relatively small, particularly males aged 50–59 years, which may limit the stability of percentile estimates within those strata. Eccentric hamstring strength was assessed using a specific Nordic hamstring testing protocol and device, which may limit direct comparability with values obtained using other assessment systems or testing procedures. Finally, injury history, resistance training history, and body composition were not included in the present analyses and may have influenced observed strength values.

Despite these limitations, the present findings provide practical reference values for clinicians, coaches, and practitioners working with older athletic populations. These data may assist interpretation of eccentric hamstring strength assessments during athlete monitoring, rehabilitation, and performance testing in senior athletes, providing context for absolute and body mass–normalized force and inter-limb asymmetry.

### 4.5 Conclusion

This study established sex-stratified reference values for eccentric hamstring strength in competitive senior athletes aged ≥50 years using instrumented Nordic hamstring testing. Male athletes demonstrated greater absolute and relative peak force than females, and increasing age was associated with lower strength across the cohort, whereas asymmetry demonstrated little association with age or sex. These findings provide practical reference values for interpreting eccentric hamstring strength assessments in older athletic populations and may assist clinicians and practitioners involved in athlete monitoring and performance evaluation.

## Data Availability

The raw data supporting the conclusions of this article will be made available by the authors, without undue reservation.
